# PAX3d mRNA over 2.76 copies/µL in the bloodstream predicts cutaneous malignant melanoma relapse

**DOI:** 10.18632/oncotarget.20177

**Published:** 2017-08-11

**Authors:** Chiara Autilio, Carmela Paolillo, Maria Michela Lavieri, Krizia Pocino, Elisa De Paolis, Enrico Di Stasio, Paolo Marchetti, Cappellini Antonini Gian Carlo, Ettore Capoluongo

**Affiliations:** ^1^ Institute of Clinical Biochemistry, Laboratory of Clinical Molecular Diagnostics, Fondazione Policlinico “A. Gemelli”, Catholic University of the Sacred Heart, Rome, Italy; ^2^ Unit of Dermatology, “Cristo Re” Hospital, Rome, Italy; ^3^ Laboratory of Clinical Biochemistry, Fondazione Policlinico “A. Gemelli”, Rome, Italy; ^4^ Sant’Andrea Hospital, Rome, Italy; ^5^ Laboratory of Advanced Molecular Diagnostics (DIMA), Istituto Dermopatico dell’Immacolata, Fondazione Luigi Maria Monti, IRCCS, Rome, Italy; ^6^ Oncology Unit Immacolata Dermatological Institute (IDI), Rome, Italy; ^7^ “Molipharma Srl” a Spinoff of Catholic University, Campobasso, Italy

**Keywords:** melanoma molecular biomarker, qRT-PCR, melanoma relapse, circulating tumor cells, PAX3

## Abstract

**Objective:**

The aim of this study was to evaluate if our molecular algorithm, based on tumor circulating transcripts, may predict relapse risk in cutaneous malignant melanoma (CMM).

**Results:**

The multi-marker panel was able to differentiate patients with CMM from HC with high diagnostic sensitivity and specificity, especially for MITF-m and TGFB2 (91–100%) whose levels decreased during follow-up of recurrence-free patients, and remained stable in the case of relapse. PAX3d higher than 2.76 copies/µL emerged as a promising biomarker [specificity = 75–93% and negative predictive value = 75–98%] to stratify subjects at high risk of CMM recurrence independently of age, gender and AJCC staging [OD = 9.5(3.2–28.0), *p* < 0.001]. The survival analysis confirmed PAX3d performance in relapse prediction with significant differences in recurrence risk 12 months after the basal time-point (*p* = 0.008).

**Materials and Methods:**

Peripheral blood was collected from 111 CMM patients and from 87 healthy controls (HC) randomly selected. Each specimen was examined by qRT-PCR analysis for the expression of 3 tumor-related transcripts (PAX3d, MITF-m and TGFB2) at diagnosis, and at the following 6 and 12 months during clinical monitoring.

**Conclusions:**

We demonstrated the usefulness of our molecular algorithm to indirectly detect circulating melanoma cells in blood, along with PAX3d capability to assess patients’ progression and relapse prediction.

## INTRODUCTION

Cutaneous malignant melanoma (CMM) is the most serious and deadliest type of cancer, in spite of only 4% of incidence among dermatological malignancies [1]. Although the early diagnosis aims at carrying out effective surgical interventions in primary tumors, the metastatic disease still causes more than 80% of deaths because of its aggressiveness and resistance to current therapies [2, 3].

Nowadays, no serological and molecular biomarkers for early-stage disease have been included in the latest American Joint Committee on Cancer (AJCC) staging and classification guidelines [4]. Indeed, AJCC suggested only serum lactate dehydrogenase (LDH) which is involved in advanced stages of CMM since LDH serum levels increase when the tumor has already spread to distant organs [5]. Therefore, there is a need to identify new approaches able to predict patients at high risk of relapse as early as possible, and thus allow for more effective therapeutic intervention [6].

As reported in literature for epithelial tumors, circulating tumor cells (CTCs) which shed from primary tumor are responsible for metastatic dissemination and clinical relapse [7]. Hence the current purpose of CMM research is to isolate CTCs from bloodstream with high levels of specificity, which may allow for their targeted capture [8–10]. Unfortunately, unlike other cancers, circulating melanoma cells (CMCs) are difficult to enrich as they do not express common CTCs markers. Moreover, the following limitations are present regarding CMCs enrichment and characterization: a) CMC cellular heterogeneity, b) the different isolation platforms available for their processing, c) the lack of adequate clinical trials. These concerns did not lead to an international consensus on CMCs clinical usefulness and application as a standard method [11, 12].

A previous study conducted by our research group [13] identified three mRNA transcripts (PAX3d, MITF-m and TGFB2) related to the presence of CMCs by using qRT-PCR. Indeed, recent reports have suggested that melanoma cells switch back to the embryogenetic program initiated during neural crest formation [14] by means of several factors as PAX3 and MITF.

The first protein is a member of the PAired boX family involved in melanoblasts development [15] and especially its isoform “d” [16] is expressed in both melanoma tissue and CMCs. MITF is another key transcription factor promoting neural crest and derivatives formation [17]. The isoform “m” is mainly expressed in CMM [18] as an essential regulator of the tumor survival and growth [19]. Similarly, especially TGFB isoform 2 plays a key role in melanoma aggressiveness [20].

Although our molecular panel differentiated healthy controls (HC) from patients with high diagnostic values of sensibility and specificity (93% and 100%, respectively), we performed the study on a small population size. Furthermore, we did not obtain any data about the prognostic significance of the three circulating transcripts, along with their relationships with metastasis spreading.

The aim of the present paper is to evaluate the prognostic and predictive values of such panel for tumor recurrence, regardless of AJCC staging. Clearly, an increase in tumor biomarkers for patients diagnosed with melanoma at IV stage can be expected, however the big challenge is to obtain a rise during the follow-up mainly in those subjects who developed tumor recurrence at II–III stages.

We have analyzed the changes in PAX3d, MITF-m and TGFB2 copy numbers on a larger CMM patients’ cohort (with or without melanoma relapse) during their clinical monitoring.

The ultimate purpose is to assess the capability of our method, in term of sensitivity and specificity, along with its usefulness for the tumor recurrence prediction, particularly at the early stage.

## RESULTS

### Clinical and pathological characteristics of patients

Table 1 shows the clinical characteristics of the subjects analyzed for PAX3d, MITF-m and TGFB2 mRNA expression. The patients’ cohort was subdivided into groups according to gender, age, recurrence site and the following prognostic features: AJCC staging (TNM I–IV and uveal melanoma), Breslow index (≤ 1.5: thin, 1.6–4: intermediate, > 4: thick or pTX: primary tumor cannot be assessed), sentinel lymph node biopsy, presence of a relapse before the patients’ recruitment or a disease recurrence during the patients’ follow-up. Ages and gender were differently distributed between CMM and HC (*p* < 0.01; *p* < 0.04, respectively). Nevertheless, tumor variables did not show significant differences when analyzed for age and gender distribution (AJCC: *p* = 0.535, *p* = 0.491; Breslow index: *p* = 0.203, *p* = 0.102; primary site: *p* = 0.345, *p* = 0.224; sentinel lymph node: *p* = 0.250; *p* = 0.090; recurrence: *p* = 0.159, *p* = 0.407; tumor relapse: *p* = 0.385, *p* = 0.240).

**Table 1:** Clinical and pathological characteristics of subjects

Prognostic features	CMM patients	HC	*p*-value
***Gender (a)***	***n* (%)**	***n* (%)**	
Male	59 (53.2 %)	35 (40.2 %)	0.040 *
Female	52 (46.8 %)	52 (58.8 %)	0.218 *
***Age (b)***	**y ± SD**	**y ± SD**	
Male	60.2 ± 14.3	38.0 ± 9.7	0.009 **
Female	53.4 ± 15.1	39.2 ± 10.8	0.008 **
***AJCC***	***n* (%)**		
I	17 (15.3 %)	-	0.491 (a)/0.535 (b) ***
II	16 (14.4 %)	-	
III	64 (57.7 %)	-	
IV	12 (10.8 %)	-	
Other	2 (1.8 %)	-	
***Breslow index (mm)***	***n* (%)**		
pTX	10 (9.0 %)	-	0.102 (a)/0.203 (b) ***
≤ 1.5	37 (33.3 %)	-	
1.6–4	33 (29.7 %)	-	
> 4	28 (25.3 %)	-	
Uveal/Mucosal	3 (2.7 %)	-	
***Primary site location***	***n* (%)**		
Back	46 (41.4 %)	-	0.224 (a)/0.345 (b) ***
Abdomen	10 (9.0 %)	-	
Lower limb	25 (22.5 %)	-	
Arm	9 (8.1 %)	-	
Head-neck	6 (5.4 %)	-	
Other	4 (3.6 %)	-	
Unknown	11 (10 %)	-	
***Sentinel lymph node***	***n* (%)**		
Positive	52 (46.8 %)	-	0.090 (a)/0.250 (b) ***
Negative	44 (39.6 %)	-	
Unknown	15 (13.5 %)	-	
***Recurrence site***	***n* (%)**		
Skin in transit metastasis and lymph nodes	9 (37.5%)	-	
Liver, lung and lymph nodes	9 (37.5%)	-	0.407 (a)/0.159 (b) ***
Brain	6 (25%)	-	
***Tumor relapse***	***n* (%)**		
Absent	63 (56.8 %)	-	0.240 (a)/0.385 (b) ***
Before enrollment	4 (3.6 %)	-	
During follow-up	24 (21.6 %)	-	
Before and during follow-up	20 (18.0 %)	-	

Abbreviations: HC = Healthy Controls; CMM = Cutaneous Malignant Melanoma; PTX = primary tumor cannot be assessed; *n* ***=*** number of subjects; y = years; SD = standard deviation.

* Mann Whitney test of genders between CMM patients and HC;

** Mann Whitney test of ages between CMM patients and HC;

*** Kruskal Wallis test of gender (a) and age (b) between clinical subclasses according to different prognostic factors.

### Basal values of mRNAs and prognostic factors

Table 2 shows the absolute copy number (copies/µL) of PAX3d, MITF-m and TGFB2: a) in all patients and controls, b) in different AJCC subgroups (uveal, I, II, III, IV), stratified by Breslow depth in mm (≤ 1.5: thin, 1.6–4: intermediate, > 4: thick or pTX: primary tumor cannot be assessed). The table shows mRNAs levels expressed as median and interquartile ranges (1st–3th). As expected significant differences, between CMM patients and HC, for all biomarkers (PAX3d: *p* < 0.0001, MITF-m: *p* < 0.0001 and TGFB2: *p* < 0.0001) were observed.

**Table 2:** mRNAs levels expressed as median and interquartile range (1st – 3th) of the 3 biomarkers, depending on clinical and pathological features

	Median (Q1-Q3) values of transcripts level (copies/µL)			
	*N*	PAX3d	MITF-m	TGFB2
***Subjects***				
HC	87	0.00 (0.00–0.10)	7.20 (2.50–19.40)	1.20 (0.60–2.50)
CMM patients	111	0.78 (0.11–1.98)	158.41 (72.89–427.16)	35.29 (11.96–85.02)
*p-value (a)*		**< 0.0001**	**< 0.0001**	**< 0.0001**
***AJCC***				
I	17	0.91 (0.00–1.90)	555.8 (300.7–721)	55.62 (28.11–106.94)
II	16	0.16 (0.0–1.22)	125.12 (68.23–232.04)	13.48 (5.91–34.78)
III	64	0.71 (0.24–1.88)	154.28 (64.04–359.30)	35.71 (11.71–93.30)
IV	12	3.32 (1.59–4.93)	122.97 (80.74–232.42)	36.42 (20.41–73.01)
Other	2	1.72 (1.51–1.92)	50.29 (43.00–57.58)	29.42 (24.34–34.49)
*p-value (b)*		**0.010**	**0.002**	0.060
***Breslow index (mm)***				
pTX	10	1.22 (0.43–2.38)	195 (61.49–398.61)	59.91 (33.61–86.73)
≤ 1.5	37	0.91 (0.05–2.27)	300.7 (104.8–577.5)	43.35 (25.26–111.04)
1.6–4	33	0.68 (0.19–1.85)	175.15 (84.57–353.88)	34.81 (13.59–69.09)
> 4	28	0.69 (0.0–1.34)	122.73 (59.46–183.83)	13.9 (6.39–39.74)
Uveal/Mucosal	3	1.95 (1.71–2.12)	70.45 (58.00–97.58)	29.34 (31.47–44.90)
*p-value (b)*		0.553	**0.021**	0.068

GAPDH and beta-actin were used as reference genes being their levels not fluctuating between patients and controls and within patients’ subgroups

Abbreviations: HC = Healthy Controls; CMM = Cutaneous Malignant Melanoma; PTX= primary tumor cannot be found; Q1= first quartile; Q3= third quartile.

(a) *p*-value was obtained by Mann Whitney *U*-test.

(b) Overall *p*-value was obtained by Kruskal Wallis test.

When patients were subdivided according to AJCC staging, the statistical significance was only achieved for PAX3d (*p* = 0.010) and MITF-m (*p* = 0.002). The first biomarker showed a tendency to increase at stage IV, whereas MITF-m mRNA levels raised mainly in stage I, with a subsequent reduction from the stage II onward. However, MITF-m amount in overall patients remained significantly higher than that observed in HC.

Likewise, when comparing MITF-m transcripts with Breslow index, the significance was observed only for thin melanoma (≤ 1.5, *p* = 0.021), which is often related to the early stages of the disease.

Surprisingly, neither PAX3d circulating levels were related to Breslow depth (R = 0.06), nor the remaining ones (MITF-m and TGFB2 mRNA): R = −0.16 and R = −0.05, respectively.

### Diagnostic performance by ROC curve analysis

**Figure 1:**
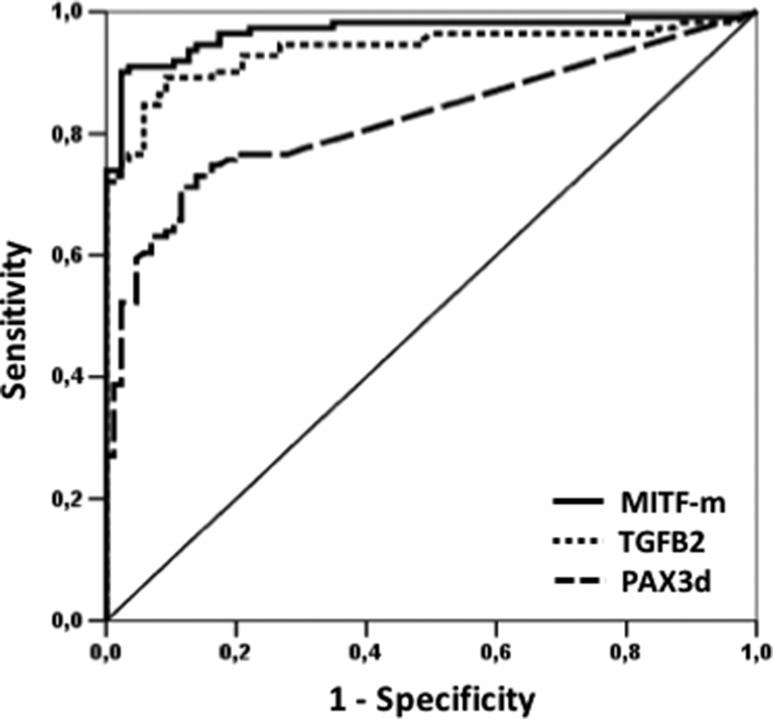
ROC curve analysis of PAX3d, MITF-m and TGFB2 in CMM patients versus HC at the time of diagnosis The solid line represents MITF-m values, whereas the lines with large and small tracts corresponded to PAX3d and TGFB2 levels, respectively.

Basal values of PAX3d, MITF-m and TGFB2 were used to confirm the diagnostic performance of our molecular panel, by comparing controls and patients’ mRNA levels using ROC curve analysis. Thereby, novel cut-off values able to discriminate HC from subjects with CMM were defined. The area under the ROC curve is depicted in Figure 1, resulting as 0.967 (95% CI: 0.942 to 0.992) for MITF-m, 0.936 (95% CI: 0.900 to 0.973) for TGFB2 and 0.823 (95% CI: 0.764 to 0.881) for PAX3d. The following mRNAs copies were selected as best cut-off values: 42.90 copies/µL for MITF-m (diagnostic sensitivity and specificity of 91% and 97%), 4.78 copies/µL for TGFB2 (89% and 100%, respectively) and 1.0 copies/µL for PAX3d (51% and 97%, respectively).

### Gene expression of biomarkers during patients’ follow-up

**Figure 2:**
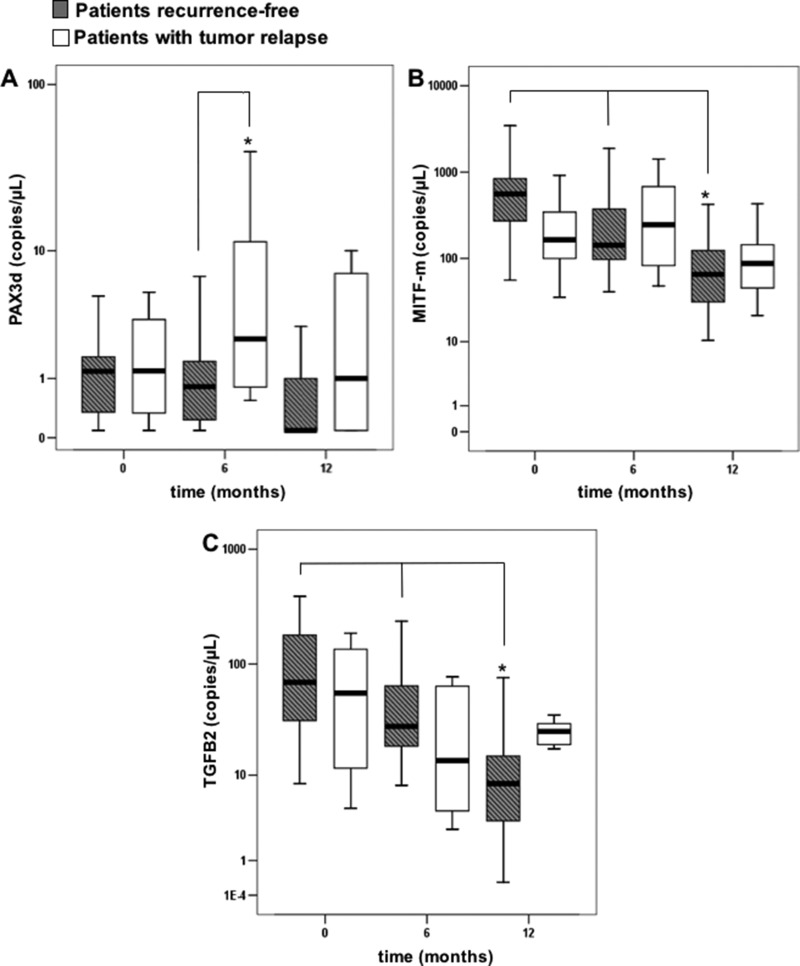
Copies of transcripts in the patients with or without tumor relapse, evaluated at basal time (T0), and during the follow-up (6 and 12 months) Errors bars represent interquartile range, whereas horizontal black bars represent median values; asterisks represent: (**A**) a significantly difference (*p*-value < 0.05) between disease-free patients and relapse patients by Mann Whitney test, (**B**, **C**) a significantly difference (*p*-value < 0.05) over time within disease-free patients by Friedman test.

In order to evaluate if the aforementioned transcripts were able to work as early stage biomarkers of relapse, mRNAs copies were monitored over 6 and 12 months after the enrollment (T0) for each patient. We divided the subjects in two subgroups: 24 patients who relapsed and 87 being recurrence free. Figure 2 shows box plots for PAX3d, MITF and TGFB2 in each subgroup at T0, 6 and 12 months. When comparing copies of each mRNA among the different follow-up time points, no significant differences for PAX3d, neither in patients without relapse [basal value: 1.20 (0.21–1.96) copies/µL, 6 months: 0.79 (0.05–1.67) copies/µL, 12 months: 0.47 (0.30–1.05) copies/µL, *p* = 0.906)] nor in patients with recurrence [basal value: 1.29 (0.23–3.90) copies/µL, 6 months: 2.42 (0.73–13.82) copies/µL, 12 months: 1.02 (0.36–8.37) copies/µL, *p* = 0.223] were found.

Contrastingly, a significant reduction in MITF-m and TGFB2 levels was observed during the follow-up of patients without disease progression [MITF-m basal value: 556.6 (257.44–846.00) copies/µL; six months: 141.9 (96.91–552.09) copies/µL; 12 months: 65.24 (30.18–129.95) copies/µL, *p* < 0.001; TGFB2 basal value: 69.09 (29.97–183.89) copies/µL, 6 months: 28.10 (18.29–74.93) copies/µL, 12 months: 8.30 (3.26–16.28) copies/µL, *p* < 0.001]. No significant decrease was observed in patients with melanoma recurrence (*p* = 0.197 for MITF-m; *p* = 0.325 for TGFB2).

When we compared the two subgroups of patients (with or without relapse) at each time point for each biomarker, a significant difference in PAX3d levels was found six months after the basal-value [patients with tumor relapse: 2.42 (0.73–13.82) copies/µL *vs* recurrence-free patients: 0.79 (0.05–1.67) copies/µL, *p* = 0.001].

**Figure 3:**
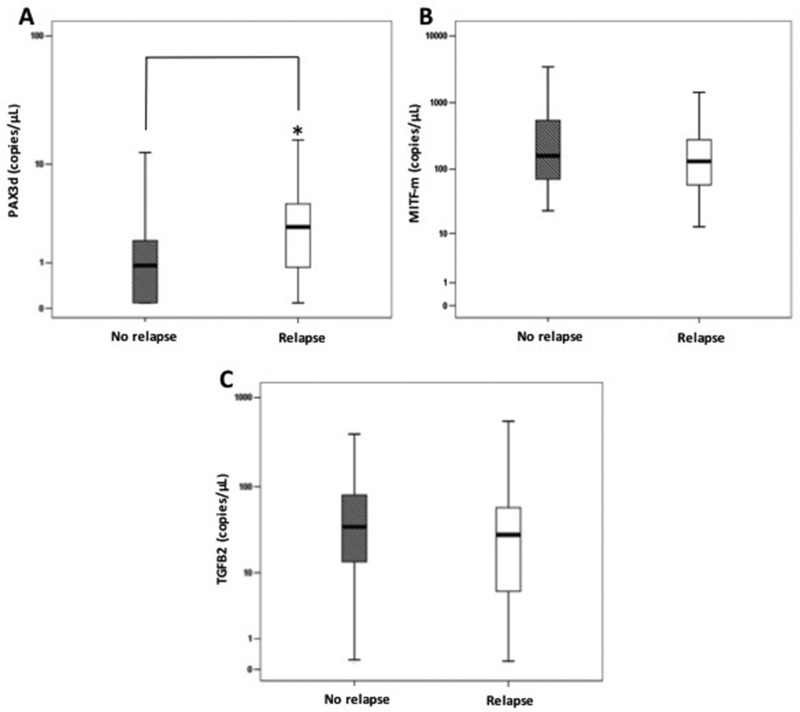
Comparisons among PAX3d, MITF-m and TGFB2 copies/µL at basal time-point (T0) and those observed 6 months before relapse in patients with or without relapse Errors bars represent interquartile range, whereas horizontal black bars represent median values. The asterisk represents: (**A**) a significantly difference (*p*-value < 0.05) between disease-free patients and relapse patients for PAX3d levels by Mann Whitney test. (**B**–**C**) No significantly difference was observed for MITF-m and TGFB2.

We also compared PAX3d, MITF-m and TGFB2 values found at basal time-point in recurrence-free patients (*N* = 87) with those assayed (*N* = 24) six months before any relapse event in all subjects with progression (Figure 3). Within the three biomarkers, only PAX3d notably increased in patients relapsing during the follow-up [PAX3d no relapse subjects: 0.91 (0.00–1.88) copies/µL, PAX3d relapse subjects: 2.71 (0.83–4.59) copies/µL, *p* = 0.041], whereas MITF-m and TGFB2 copies did not significantly change in the two clinical conditions [MITF-m no relapse subjects: 158.4 (70.4–540.2) copies/µL, MITF-m relapse subjects: 128.6 (61.7–260.5) copies/µL, *p* = 0.475; TGFB2 no relapse subjects: 34.8 (13.6–80.5) copies/µL, TGFB2 relapse subjects: 28.2 (5.7–58.9) copies/µL, *p* = 0.857]. However, PAX3d levels are not significantly correlated to metastatic site in patients with tumor relapses (*p* = 0.891).

### PAX3d as predictive biomarker for relapse

To better understand the predictive role of PAX3d whose only transcripts increase during the patients’ monitoring, we selected the 90th percentile of basal values in subjects without relapses (*n* = 87, 2.76 copies/µL) as a molecular cut-off for recurrence prediction. Since we obtained a similar value (*n* = 261, 2.65 copies/µL) at the 90th percentile for transcripts assayed throughout the entire period of follow-up (at basal, six and twelve months of observation), we fixed at 2.76 copies/µL the reference value. As expected, both values are significantly higher than that observed in HC (*n* = 87, 0.51 copies/µL; *p* < 0.0001).

To investigate if the fixed cut-off (PAX3d = 2.76 copies/µL) was able to predict the risk of relapse, a ROC curve analysis was carried out, by comparing copies basal values of patients who were recurrence-free (*N* = 87) to those of patients with a relapse event at stage II–III (*N* = 18) or at stage IV (*N* = 6). In these two subgroups, we considered PAX3d values obtained six months before the tumor recurrence. The analysis pointed out for relapse-patients at IV stage a specificity for prognosis of 93% and a sensitivity of 75% (PPV and NPV of 43% and 98%, respectively). At the same time, for relapsing II–III stage patients, we obtained a specificity for prognosis of 75% and a sensitivity of 67% (PPV and a NPV of 67% and 75%, respectively).

By using a multivariate logistic analysis, we observed as the copies of PAX3d over cut-off were predictive of relapse regardless of age, gender and AJCC stages [Odds Ratio (OD) of 9.5 (3.2–28.0), *p* < 0.001].

**Figure 4:**
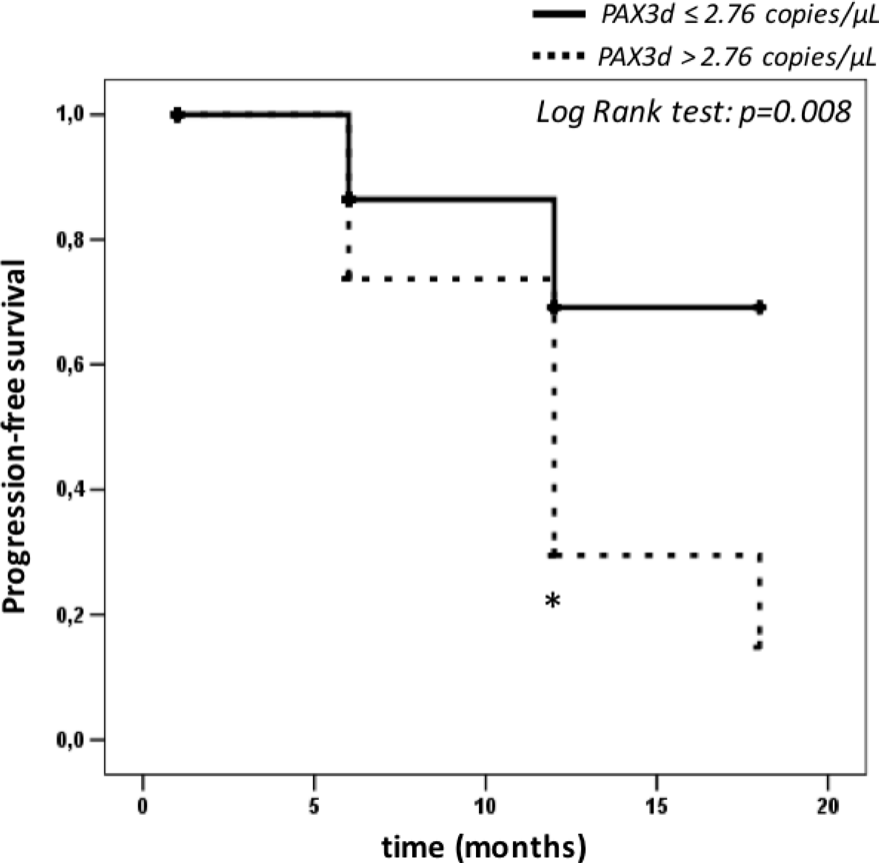
Kaplan-Meier survival curves calculated on patients with relapse (*n* = 24), depending on PAX3d cut-off value of 2.76 copies/µL The asterisk represents: a significantly difference (*p*-value < 0.05) between relapse patients with PAX3d values above > 2.76 copies/µL (*n* = 12) and PAX3d values ≤ 2.76 copies/µL (*n* = 12) by Log Rank test.

Furthermore, we carried out a Kaplan-Meier analysis by evaluating the risk of relapse associated to PAX3d values (above or below the cut-off of 2.76 copies/µL) over 18 months of follow-up (Figure 4). The analysis underlined a considerable difference between the risk scores obtained by the two groups (group 1 = patients with PAX3d values ≤ 2.76 copies/µL; group 2 = patients with PAX3d values < 2.76 copies/µL). Such result was observed 12 months (*p* = 0.008) after the patients’ enrollment, and showed an evident decrease in “progression free rate” above all in those with PAX3d copies > 2.76 copies/µL at 6 months-time-point (Figure 4).

## DISCUSSION

CMM accounts for a small percentage of all skin malignancies, but it is still responsible for a majority of deaths due to cutaneous cancers [21]. In 2017, the overall 5-year survival in patients with very early-stage disease was estimated as being over 98%, with a decreasing when the tumor has spread to the nearby lymph nodes or to distant organs (reaching 62% and 18%, respectively) [22].

AJCC guidelines still suggested the clinical and histopathological classification at diagnosis as the only prognostic factors, along with LDH detection during the follow-up of patients at stage IV of the disease [4]. However, although clinical observations provide an accurate prognosis for the most part of patients, they frequently fail to identify subjects who develop relapses in the early stages of melanoma [23]. Moreover, although LDH is reported as a surrogate biomarker of CMM invasion in patients at stage IV of the disease, various inflammatory, ischaemic and infective processes may increase its levels [5]. Thus, serum LDH levels are not specific for CMM and tend to increase late when metastases have already spread to distant organs [5].

Therefore, the main challenge for clinicians is to develop novel biomarkers which may predict a tumor recurrence as early as possible in order to promote rapid therapeutic interventions. However, the most recently proposed putative prognostic markers need to be clinically validated on a large scale and eventually do not add useful information to AJCC classification [24–28].

As reported in literature, pharmacological treatments for metastatic cancer are more efficacious at early-stages (M1a/M1b) rather than when distant formations have already been found (M1c) [29, 30]. Hence it is crucial to identify the early stages of melanoma progression when tumor cells undergo a mesenchymal–epithelial transition for their extravasation into bloodstream [31, 32].

Because of the limitations of current CMC-isolating methods [12], the liquid biopsy of other circulating tumor elements has been suggested as a useful tool for relapse prediction. However, circulating tumor-DNA and microRNAs are relatively resistant and may also originate from dead cancer cells [33]. On the contrary, once released, tumor mRNAs are instable and their detection in bloodstream is related to the viable presence of CMCs. In this context, the main purpose is to select expression markers which can reflect metastatic progression.

Recent reports have suggested that melanoma cells switch back to the embryogenetic program initiated during neural crest formation [14] by means of several factors as PAX3 and MITF [15, 16, 19, 34–36].

During embryogenesis the two proteins are interrelated [37]: Pax regulates MITF expression to steer progenitor cells in melanocytic lineage. However, once the melanocytes development is completed, the PAX3d-growth effect is inhibited by TGFB [38, 39]. Conversely, in CMM cells TGFB has a different role as it appears involved in the tumor aggressiveness, angiogenesis, cells growth, migration and regulation of immunological surveillance [23, 40]: most of these mechanisms seem to be also associated to PAX3d and MITF expression [23, 34, 35].

Based on these considerations, the aim of our work was to confirm in a larger cohort of patients the diagnostic performance of PAX3d, MITF-m and TGFB2 circulating tumor transcripts for CMM detection. We can speculate that such transcripts are correlated to viable CMCs and come from cellular components because we extracted total RNA after a centrifuge at 2000 g for 5 minutes, discarding the supernatant according to manufacturer instructions. Hence, considering that weak centrifugations are able to precipitate only cellular components (CTCs, leucocytes, epithelial cells and large vesicles) we missed the major part of other tumor derivatives (exosomes and vesicles ranging from 100 to 150 nm). Indeed, as reported in literature, to precipitate such components it’s necessary a more powerful centrifugation of 10,000 g [41].

Moreover, we evaluated a possible correlation between melanoma relapse and the increase in copies of the above described three transcripts during the patients’ twelve months follow up.

As in our previous study, quantitative data expressed as copies/µL of the three biomarkers were significantly higher in CMM patients than in HC [13]. ROC curve analysis corroborated the results already described for MITF-m with high diagnostic sensitivity and specificity values, albeit slightly lower when compared to the previous ones: 91% and 97% against 100%. The best diagnostic cut-off was similar to that reported in our previous study: 42.90 *versus* 34.43 copies/µL.

Regarding PAX3d, the diagnostic specificity remained unchanged, whereas the diagnostic sensitivity clearly decreased from 93% to 51%, although in presence of a cut-off value not significantly different from the previous one: 1.00 copies/µL compared to 1.23 copies/µL [13]. This result may be related to the different cohort of patients selected in the present work which now includes subjects at stage I of disease. Therefore, we can speculate that such decrease in specificity is due to similar values in PAX3d between HC and patients at stage I of melanoma. Indeed, we cannot exclude that the lowest levels of PAX3d are due to: a) the increased number of healthy volunteers enrolled in this study (87 against 30 of the previous paper), b) the inclusion of stage I CMM patients (17 *vs* “0”) [13]. Conversely, the best cut-off maintained high diagnostic sensitivity and specificity values for TGFB2 (89% and 100%), while the copy number dropped from 37.15 copies/µL to 4.78 copies/µL, probably due to the rising number of HC individuals (from 30 to 87 subjects).

These results confirm as: a) MITF and TGFB2 can adequately distinguish HC from CMM patients, b) the accuracy of our molecular algorithm in identifying CMCs in patients’ bloodstream. In regard to PAX3d, although with a lower diagnostic sensitivity, it can still be considered as a key biomarker for relapse prediction, since it resulted as the only transcript that significantly increased in advanced stages of disease (III and IV).

When we divided patients into AJCC classes, which reflect tumor aggressiveness, a different behavior of the 2 biomarkers MITF-m and PAX3d was observed. MITF-m levels, as for PAX3d, were higher in CMM patients than HC. However, when we analyzed its amount depending on AJCC staging at diagnosis, an opposite tendency was evident: MITF-m mRNA seems to increase for patients at stage I of CMM, whereas it decreases from stage II onward, although remaining higher than in HC. To confirm such result, we also observed a significant rise in MITF-m expression in thin tumors (< 1.5 mm) when compared to thicker melanoma.

Literature evidences have already demonstrated a) the presence of tumor transcripts in patients at stage I of melanoma [42], b) a wide *in vitro* variability in MITF-m levels depending on melanoma cell lines migration (low MITF-m expression cell lines have a migration rate twenty-three times higher than the high MITF-m expression ones) [43, 44], c) a reduction *in vivo* MITF levels in melanoma tumors of patients with high mortality rate compared to those observed in mild forms of CMM [45].

In definitive, MITF is proposed to modulate cell activity through variable expression just as a rheostat that alters resistance in a circuit [46, 47]. At high levels, the transcription factor promotes cellular proliferation [45], leading also to a less aggressive phenotype related to the *miR-211* modulation with the consequent reduction in POURF2 transcripts [15]. On the contrary, lower levels of MITF (although higher than the same ones observed in healthy controls) cause an increase of Rho-associated protein kinase (ROCK) activity, which promotes cellular migration [45].

However, apart from higher MITF-m levels at diagnosis of disease stage I, the overall tendency during the follow up of recurrence-free patients was a reduction in MITF-m expression. MITF-m and TGFB2 transcripts within relapse-free patient group were significantly reduced twelve months after the basal time-point (reaching almost the HC levels), whereas PAX3d amount did not vary overtime. Such result highlights how the non-visible reduction in MITF-m and TGFB2 levels during patients’ follow-up can be considered as a warning of disease progression risk. On the contrary, when PAX3d levels were analyzed during the follow-up within the two subgroups (both patients with and without relapse), no changes in its amount were found.

Moreover, when only subjects with tumor recurrence were considered, a significant increase of PAX3d was evident, particularly six months after their enrollment, while MITF-m and TGFB2 levels did not change. Noteworthy, disease recurrence occurred immediately before or a few months after this time-point. To corroborate our result, we also compared basal values of PAX3d, MITF-m and TGFB2 obtained in recurrence-free patients to those observed six months before melanoma relapse in subjects with a disease progression.

Recurrence was considered as a disease worsening based on clinically and/or histologically evidences which were confirmed as a loco-regional or at distance tumor lesions.

Among the three transcripts, PAX-3d significantly increased, therefore confirming that it is a promising biomarker for CMM recurrences already at early stages of tumor progression

Interestingly, literature evidences carried out to date are not in contradiction with our results: Eccles el al. [15] suggested the theory of “genetic switch”, as they described how PAX3-POU3F2 and MITF-miR-211 contribute independently to phenotypic fate of melanoma cells. The model provides that, although melanoma cells switch back to their embryogenetic program, PAX3 in CMM does not modulate MITF expression as it accounts for neural crest formation [43]. As the melanoma invasiveness intensifies, CMCs exhibit high amount of PAX3 transcripts which leads to a reduction in pigmentation and mitotic rate as well as an increase in cellular migration [15].

When we set 2.76 copies/µL of PAX3d as progression cut-off value, ROC analysis pointed out a high specificity and NPV in recurrence prediction both for relapse-patient at II–III stages (75% and 75%, respectively) and IV stages of melanoma (93% and 98%). Therefore, we demonstrated the prognostic performance of PAX3d as a predictive cut-off both in early disease and advanced stages, underling how its values can predict relapses regardless to AJCC staging.

However, we obtained a lower sensitivity and PPV. Such reduction in sensitivity may be due to the small size of patients with relapse (*n* = 18 and 6) in our cohort: this condition forced us to calculate our prediction cut-off, starting from the 90th percentile of recurrence-free subjects who were a higher number than the ones in the other subgroup (87 against 24). Nevertheless, our results are promising, considering that the diagnostic goal in tumor recurrence is to identify true positives – namely patients at high risk of relapse.

Finally, we also showed as PAX3d values higher than 2.76 copies/µL can stratify patients at high risk of CMM recurrence independently of age, gender and AJCC staging. The survival analysis confirmed the robustness in relapse prediction of such biomarker depending on PAX3d cut-off. In this context, values more than 2.76 copies/µL may help clinicians to start intensive clinical monitoring in CMM patients at high risk of disease.

Up to now, LDH is the only marker suggested as a predictor of melanoma progression by AJCC guidelines, merely in patients with stage IV of the disease [4]. On the contrary, we showed as PAX3d is an earlier biomarker being over cutoff at stage II and III.

Unfortunately, we followed patients during a period of 18 months: therefore, we cannot provide more information about the long-term prediction ability of our molecular algorithm. We are aimed to continue with the follow-up of the remaining high stage melanoma patients, also including new individuals, above all considering that melanoma can relapse many years later the surgical excision of primary lesion.

To conclude, our multi-marker panel can be confirmed as an useful surrogate test for minimal residual disease evaluation, since it allows for an early diagnosis of metastatic development and tumor relapse. We decided to elaborate a molecular algorithm whose methodological processes could be suitable for clinical practice, by using a commercially available method for RNA extraction coupled to qRT-PCR technique. The latter provides some advantage being cost saving quick and easy to use: this will facilitate the introduction of this type of assay in routine practice for overall CMM patients.

## MATERIALS AND METHODS

### Subjects

We enrolled 111 patients divided into 59 men (between 22 and 83 years of age) and 52 women (between 12 and 79 years of age) after histopathological diagnosis of CMM. All subjects were examined for over 18 months. We collected patients’ blood samples immediately after melanoma excision which was considered the basal time-point (T0) and, subsequently, at the following 6 and 12 months during clinical monitoring. Therefore, we have two different monitoring: the first corresponds to 18 months of clinical follow-up, whereas the second refers to tumor-transcripts detection during 12 months after patients’ enrollment. For each patient, the following data were collected: numbers of melanoma, Breslow index, TNM and number of metastasis. The clinical classification of patients was carried out at Immacolata Dermatological Institute according to the latest AJCC guidelines [4], by a trained oncologist.

The subjects were classified as follows: 2 patients with uveal melanoma, 17 with stage I, 16 with stage II (10 IIB and 6 IIC), 64 with stage III (13 IIIA, 27 IIIB, 24 IIIC) and 12 with stage IV of melanoma. Lymph node involvement was unknown in 15 patients, whereas it was negative in 44 subjects and positive in 52 patients (1 at stage II, 43 at stage III and 8 at stage IV).

During the follow-up over 18 months, 24 subjects developed a relapse which was independent of clinical staging (2 were diagnosed with stage II, 16 with stage III and 6 with stage IV).

Regarding recurrence, it was related to local and at distance tumor progression: a) subjects at stage IV with a new metastatic relapse in brain; b) patients at stage III showing a distant spread of disease in liver, lung and lymph nodes; c) subjects at stage II with a loco-regional spread of the disease or a lung metastasis.

In order to confirm the reduction in basal levels of our biomarkers in subjects without any cancer disease, *N* = 87 consecutive healthy volunteers were enrolled: 35 men (between 18 and 63 years of age) and 51 women (between 18 and 65 years of age). Obviously, none of the control subjects had a history or clinical of skin cancer or was under any treatment for other types of malignant diseases.

Both patients and healthy volunteers provided a written consent for the inclusion in this study and the investigational protocol was made by following the Helsinki criteria for research studies.

### Sample processing, RNA extraction, c-DNA synthesis

For each patient, 18 mL of blood were collected into 2 EDTA tubes of 9 mL. To avoid false positive results due to the possible transit of epithelial cells into the collected blood sample, the first tube was discarded and the analysis was performed by using the second one. Each sample was immediately stored at +4°C and processed within the first 4 hours from the blood drawn.

Total RNA was extracted from 3 mL of collected sample by using QIAmp RNA blood mini kit (Qiagen, 153 Hilden, Germany) with a DNAse incubation of 15’ (Qiagen RNAse-free DNAse set) and was kept frozen at −80°C until analysis. Qiagen kits are often used for the evaluation of tumor-mRNA expression [13, 42, 48] due to its high accuracy and repeatability which avoid the need to test all samples in parallel. We also underline as fresh sample processing results as better than freezing and thawing, above all when referred to RNA molecular assay.

The integrity and amount of total RNA was carried out by capillary electrophoresis with high-sensitivity “Experion chip” (Biorad, Hercules, CA). The average amount of each sample was around 70 ng/mL in a total volume of 60 mL and presented the 260/280 ratio between 2.0 and 2.2, which confirmed the absence of protein contamination. Then, about 300 ng of total extracted RNA was used to synthetize c-DNA by using Transcriptor First Strand cDNA synthesis kit (Roche Applied Science, Indianapolis, IN), as previously described [13].

### Quantitative real-time amplification

The amplification in qRT- PCR was performed by using Taqman technology on Roche Light Cycler 480 [13]. In order to generate standard curves, the cDNA of UACC257 cell line was amplified by PCR using the specific cloning primers for PAX3d, TGFB2, MITF-m and GAPDH transcripts [PAX3-EcoRI-F: 5′-TAGAATTCTACCTCATCAGCCCCAGACT-3′; PAX3-XhoI-R: 5′-CTCTCGAGACTCTCCTTTGTCTCCTATTGGG-3′; MITF-m-EcoRI-F: 5′TAGAATTCATGCTGGAAATGCTAGAATATAATCA-3′; MITF-m-XhoI-R: 5′ CTCTCGAGGCTTCAGACTCTGTGGGAAAAATAC-3′; TGFB2-EcoRI-F: 5′-CAGAATTCGCTGCACTTTTGTACCATCTAA-3′; TGFB2-XhoI-R: 5′-CACTCGAGTCATTGTCATTTTGGTCTTGC-3′; GAPDH-HindIII-F: 5′-ATAAGCTTTCTTCCAGGAGCGAGATCCC-3′; GAPDH-BamHI-R: 5′ CCTGGATCCTTGTCATACCAGGAAATGAGCTT-3′]. The amplified products were digested with HindIII (5′) and BamHI (3′) enzymes (New England Biolabs Hitchin, UK) for GAPDH target and with EcoRI (5′) and XhoI (3′) (Biolabs) for PAX3d, TGFB2 and MITF-m targets. Subsequently, each product was ligated to a pcDNA3+ vector (Invitrogen, Carlsbad, CA) previously digested with the same enzymes, using T4 DNA Ligasi (1 μl) and T4 Buffer (10×) supplied by Biolabs. Ligated vectors were transformed into Escherichia coli DH5α cells (Invitrogen), and each plasmid DNA was isolated from recombinant clones using Plasmid Maxi kit (Qiagen). Subsequently, we performed the titration of our constructs by using a quantitative assay of dsDNA, based on real-time PCR measurement of fluorescence due to the interaction of PicoGreen dye with dsDNA. We applied a Quan-IT Pico Green assay (Invitrogen) in LightCycler 480 instrument (Roche) [13]. The averaged fluorescence values were converted into DNA amounts using a calibration curve prepared with λ-DNA standard supplied by kit. The DNA concentrations were determined and the corresponding copy numbers were calculated. Serial 10-fold dilutions from recombinant plasmids were used as standard curves, each containing a known amount of input copy number in the range of 10^1^ to 10^8^ copies/μl.

The amplification from patients’ cDNAs, was carried out as previously described by using the same primers and probes [13] [PAX3d-F: 5′-AGTCTGCCAACATCTCAGTC-3′; PAX3d-R: 5′-CCCAACAAAAGGGTAATTTT-3′ ; PAX3d/i-Hyb: 5′-FAM/CCCTGTTTCTGGTCTTCGCA/TAMRA-3′ ; MITF-m-F: 5′-GATCTTTATGGAAACCAAGG-3′; MITF-m-R: 5′-TCAGACTCTGTGGGAAA-3′; MITF-m-Hyb: 5′-FAM/CAGCCAACCTTCCCAACATA/TAMRA-3′; TGFB2-F 5′-TGCTTTAGAAATGTGCAGGA-3′ ; TGFB2-R 5′-GATGCTTCTGGATTTATGGT-3′; TGFB2-Hyb 5′-FAM/CCAAAGGGTACAATGCCAAC/TAMRA-3′; GAPDH-R 5′-GTCTTCTGGGTGGCAGTGATG-3’; GAPDH-F 5′-GCACCACCAACTGCTTAGCA-3′; GAPDH-Hyb 5′-FAM/TCGTGGAAGGACTCATGACCACAG/TAMRA-3′]. We used GAPDH as housekeeping gene for quantitative analysis as previous described [13] and its levels always resulted about 1.0–1.5·10^5^ copies/µL in both patients and controls enrolled, except for some cases which were excluded from analysis. Moreover, at the beginning of our study, a second housekeeping gene (beta-actin) was also included to verify mRNA quality [49] (data not shown).

Despite of our results, some evidences describe the wide variability on values of reference genes such as GAPDH [50] in melanoma: we decided to use as normalizing factor, the amount of 25 ng of cDNA equivalent/sample (used for the preparation of qRT-PCR reaction) as previously described [13].

### Statistical analysis

We applied Kolmogorov-Smirnov test to determine whether the population was normally distributed and we obtained a non-parametric distribution. Mann-Whitney *U* test and Kruskal-Wallis ANOVA tests were performed to evaluate significant differences among patients and controls, by analyzing the whole data or by dividing subjects according to Breslow depth and AJCC staging. Spearman or Pearson coefficients were calculated, as appropriate, for correlation analysis.

Friedman test was carried out to compare the levels of circulating transcripts during the follow up.

ROC curve analysis [51] was performed to define the best diagnostic cut-off values for the three biomarkers as well as the prognostic specificity, sensitivity, PPV and NPV of PAX3d (by using as cut-off value its 90^th^ percentile in patients without relapse).

The multivariate logistic analysis was conducted to show the true prognostic value of PAX3d [52].

Kaplan Meier curves [53] were performed to evaluate how the predictive cut-off value of PAX3d could estimate the percentage of relapse risk in patients who developed melanoma recurrence.

All data were analyzed by employing the SPSS 20.0 software (Chicago, IL, 239 USA) and *p* < 0.05 was considered to indicate a statistically significant result.
